# Real-time PCR detection of *Plasmodium *directly from whole blood and filter paper samples

**DOI:** 10.1186/1475-2875-10-244

**Published:** 2011-08-19

**Authors:** Brian J Taylor, Kimberly A Martin, Eliana Arango, Olga M Agudelo, Amanda Maestre, Stephanie K Yanow

**Affiliations:** 1 Department of Oncology, University of Alberta, Edmonton, Canada; 2 Grupo Salud y Comunidad, Faculty of Medicine, Universidad de Antioquia, Medellín, Colombia; 3 School of Public Health, University of Alberta, Edmonton, Canada; 4 Provincial Laboratory for Public Health, Edmonton, Canada

## Abstract

**Background:**

Real-time PCR is a sensitive and specific method for the analysis of *Plasmodium *DNA. However, prior purification of genomic DNA from blood is necessary since PCR inhibitors and quenching of fluorophores from blood prevent efficient amplification and detection of PCR products.

**Methods:**

Reagents designed to specifically overcome PCR inhibition and quenching of fluorescence were evaluated for real-time PCR amplification of *Plasmodium *DNA directly from blood. Whole blood from clinical samples and dried blood spots collected in the field in Colombia were tested.

**Results:**

Amplification and fluorescence detection by real-time PCR were optimal with 40× SYBR^® ^Green dye and 5% blood volume in the PCR reaction. *Plasmodium *DNA was detected directly from both whole blood and dried blood spots from clinical samples. The sensitivity and specificity ranged from 93-100% compared with PCR performed on purified *Plasmodium *DNA.

**Conclusions:**

The methodology described facilitates high-throughput testing of blood samples collected in the field by fluorescence-based real-time PCR. This method can be applied to a broad range of clinical studies with the advantages of immediate sample testing, lower experimental costs and time-savings.

## Background

The availability of sensitive diagnostic tools for malaria is critical to ensure appropriate treatment for patients and to preserve the lifespan of effective anti-malarials. In the field, the most common tools for malaria diagnosis are microscopy and rapid detection tests (RDTs), which are performed directly from the blood sample. Molecular methods that amplify and detect *Plasmodium *DNA using specific reagents and platforms, such as real-time PCR, provide far greater sensitivity, but are not yet usable at the point-of-care. However, these methods have important applications in clinical research studies that involve the analysis of blood samples collected in the field, including genotyping parasite populations and monitoring drug resistance, genetic characterization of vaccine candidates, anti-malarial efficacy trials and surveillance programs [1-3].

The performance of molecular tests largely depends on the quality of the parasite DNA. Highly purified DNA requires laborious sample processing and costly reagents, kits or equipment, whereas cruder extraction methods often produce DNA that is insufficiently pure for downstream testing. The presence of PCR inhibitors from the blood, such as haemoglobin, reduces the efficiency of the molecular reaction and compromises sensitivity [4,5]. However, the discovery of DNA polymerases that are resistant to PCR inhibition enables DNA to be amplified from blood without prior extraction. For malaria, this was recently demonstrated using the Phusion™ enzyme which amplifies DNA by nested PCR directly from dried blood spots on filter papers [6].

One of the major advances in molecular diagnostics is the integration of fluorescence-based detection of DNA in real-time PCR. This poses a new challenge to direct PCR from blood as fluorophores are quenched in the presence of haemoglobin. Amplification can be achieved, but the product is not detected. One approach to overcome the quenching effect uses inhibitor-resistant *Taq *mutants in combination with an enhancer cocktail within the PCR master mix for optimal amplification and fluorescence detection [7,8]. With these reagents, real-time PCR can be performed even with 25% blood volume in the PCR reaction [8]. The usefulness of this method was evaluated for the direct detection of *Plasmodium *DNA by real-time PCR from raw patient samples and from dried blood spots collected in the field.

## Methods

### Samples

DNA for optimization of the PCR from blood was purified from *Plasmodium falciparum *3D7 *in vitro *culture [9] using DNAzol according to the manufacturer's protocol (Invitrogen Life Technologies, Carlsbad, USA). Parasite gDNA was spiked into negative blood. Negative samples (n = 7) were collected from healthy volunteers with no recent history of travel to malaria endemic areas. Blood samples from febrile patients with suspected malaria (n = 67) were obtained from the Provincial Laboratory for Public Health in Edmonton, Canada, between 2008 and 2011 following diagnosis by microscopy and routine testing by real-time PCR [10]. Of these samples, 57 were smear positive with parasitaemias ranging from < 0.01% to 9.2%; 25 of the samples had a parasitaemia < 0.1%. Microscopy was performed in regional laboratories and parasitaemias were determined from the analysis of thin smears. Two of the smear-negative samples were positive by RDT. Samples from refugees (n = 25) were collected within two weeks of arrival in Canada as part of a separate research study. All subjects were asymptomatic for malaria. Samples were first screened by microscopy and tested retrospectively by real-time PCR as reported [11]. Of the total clinical samples tested, the following species were detected by real-time PCR: *Plasmodium falciparum *(n = 39), *Plasmodium vivax *(n = 23), *Plasmodium ovale *(n = 9), and *Plasmodium malariae *(n = 2). Blood samples were collected in EDTA or citrate tubes, stored at -20°C and thawed at 4°C prior to testing. Genomic DNA from *Plasmodium knowlesi *was obtained from MR4.

Dried blood spots were prepared on 3 MM paper (Whatman) from blood samples collected from patients who attended the malaria clinic at Puerto Libertador, in Cordoba, Colombia between 2008 and 2010. Filter papers were dried at ambient temperature in the field, shipped to Medellín and stored in plastic bags at -20°C. Positive patients were symptomatic for malaria and had infections ranging from 120-39,920 parasites/μL (median value of 4763 parasites/μL) by microscopy performed on thick smears in the field. To confirm the presence of *Plasmodium *DNA, DNA was extracted using Chelex ^® ^100 (Sigma) and tested by nested PCR according to the protocol described by Snounou *et al *[12]. Of the 48 samples, 20 were positive for *Plasmodium falciparum*, 18 were positive for *Plasmodium vivax *and 10 were negative by microscopy and nested PCR. For real-time PCR, 1.5 mm diameter blood spots were excised from filter papers using a sterilized hole punch. Spots were tested directly or soaked first for 3 minutes in sterile distilled water at 50°C. This study was approved by the Health Research Ethics Board at the University of Alberta and Comité de Bioética, Sede de Investigación Universitaria, Universidad de Antioquia (Acta 07-32-126) in Colombia.

### Real-time PCR and melt curve analysis from blood

PCR was performed as described [8] in a 20 μL reaction containing 1× PCR Enhancement Cocktail (PEC-1), 1× Omni Klentaq buffer, 0.3 μL of OKT polymerase (all from DNA Polymerase technologies, St. Louis, USA), 200 μM dNTPs, 0-40× SYBR^® ^Green (Invitrogen Life Technologies, Carlsbad, USA), and blood at 5-10% of the total reaction volume. To detect the 18S rRNA gene from all species of *Plasmodium*, primers reported by Kamau et al [13] (forward 5'-GCTCTTTCTTGATTTCTTGGATG-3' and reverse 5'-AGCAGGTTAAGATCTCGTTCG-3') were used at 200 nM concentrations. Published primers were used for species-specific real-time PCR detection of *Plasmodium falciparum *(5' PLF, 3'FAR; [14]), *Plasmodium vivax*, *Plasmodium ovale *and *Plasmodium malariae *(rVIV, rOVA, and rMAL primers; [12,15]). A new *Plasmodium ovale *reverse primer (Po260; 5'-GGCAAATGCTTTCGCAGTTGT-3') with reduced primer-dimer interactions was substituted for the rOVA reverse primer. Primers were used at 400 nM except the *Plasmodium malariae *set, which was used at 800 nM. Real-time PCR was performed on the ABI 7500 Fast system (Applied Biosystems, USA) with the following conditions: 95°C for 10 min, 40 cycles of 95°C for 30 sec, 58°C for 1 min and 72°C for 1 min. This was followed by the default dissociation cycle for melt curve analysis.

All samples were tested in triplicate. For whole blood samples, the baseline was set to cycles 3-15 and the threshold was set to 20,000 fluorescence units. Samples were considered negative if the cycle threshold (Ct) value was greater than 35 or if the melt curve did not align with the positive control. For dried blood spots, the baseline was set to 5-15 and the threshold was set to 250,000 to overcome the high background fluorescence from the filter paper itself. The amplification curves generated were sometimes shallow with some variability among replicates. Samples were considered positive when at least two of the three replicates had amplification curves that crossed the threshold before cycle 35 and had the expected melt curve profile. Panel samples were tested in a blind fashion.

## Results

### Optimization of fluorescence detection from blood

**Figure 1:**
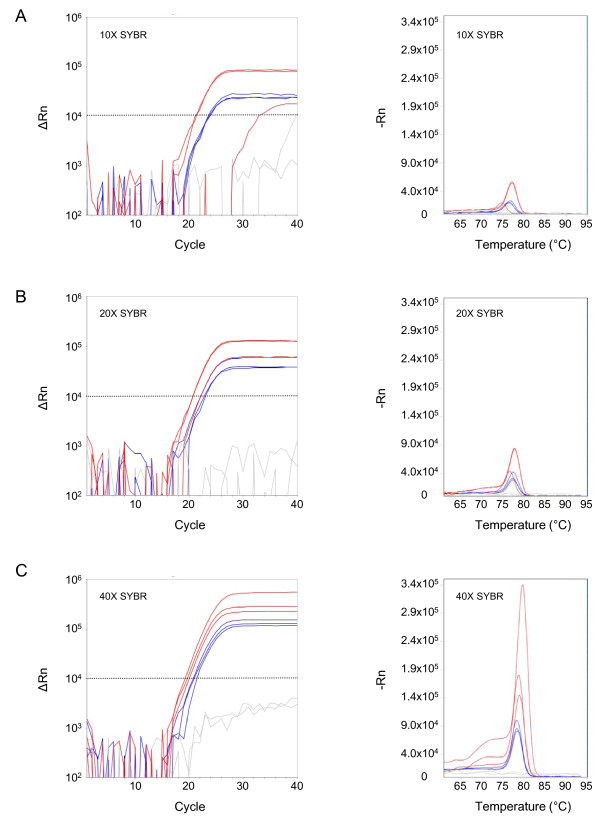
**Optimization of reagents for real-time PCR**. Amplification curves (left panels) and melt curves (right panels) from *Plasmodium falciparum *gDNA spiked into uninfected blood and tested by real-time PCR. SYBR^® ^Green dye concentrations of 10× (A), 20× (B), and 40× (C) were tested with 5% (red) and 10% (blue) volumes of blood in the PCR reaction. Uninfected blood serves as a negative control (gray). The stippled line marks the threshold for the amplification curves. Samples were run in triplicate.

A titration of SYBR^® ^Green dye was tested to determine the optimal concentration for detection of *Plasmodium *DNA directly from blood. PCR master mix with final concentrations of 10×, 20× and 40× SYBR^® ^Green was added to blood spiked with gDNA from *Plasmodium falciparum *3D7. Two blood volumes were tested: 5% or 10% of the PCR reaction volume (Figure 1). Amplification was detected at all three concentrations of SYBR^® ^Green but there was less variation in the replicates with the 40× SYBR^® ^Green concentration (Figure 1, left panels).

The melt curve profiles were also affected by the concentration of SYBR^® ^Green in the reaction. With increasing concentrations of dye, peak heights increased, thereby improving detection of the PCR product (Figure 1, right panels). At 40× SYBR^® ^Green, the melt curves were most consistent for the different blood volumes and had the highest peak heights. The fluorescence in the negative control (uninfected blood) was negligeable at this concentration. In subsequent experiments, 40× SYBR^® ^Green and 5% blood volume were selected as the optimal reaction conditions.

### Detection of *Plasmodium *from clinical samples

**Figure 2:**
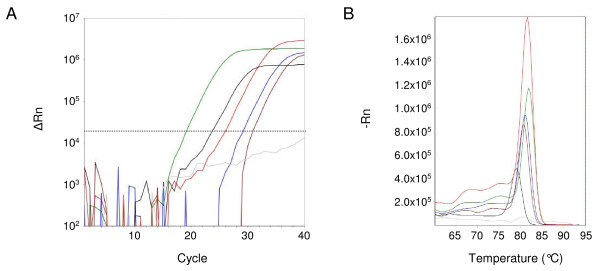
**Direct amplification from clinical samples**. (A) Representative amplification curves from clinical samples of patients with *Plasmodium falciparum *(green), *Plasmodium malariae *(black), *Plasmodium ovale *(blue), *Plasmodium vivax *(red), uninfected blood spiked with gDNA from *Plasmodium knowlesi *(brown), and uninfected blood as a negative control (gray) with the *Plasmodium *screening primers. The stippled line marks the threshold for the amplification curves. (B) Melt curves from the real-time PCR reactions in (A).

**Figure 3:**
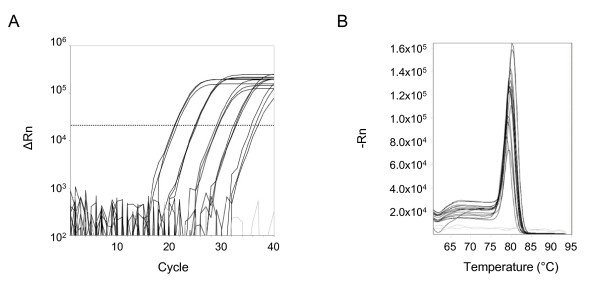
**Template dilution in blood**. Genomic DNA from a patient sample with *Plasmodium falciparum *was serially diluted ten-fold in water and spiked into whole blood for direct PCR. Amplification curves (A) and melt curves (B) of reactions performed in triplicate using the *Plasmodium *screening primers. Uninfected blood served as the negative control (gray). The stippled line marks the threshold for the amplification curves.

The reaction conditions were next assessed on whole blood samples from patients infected with malaria. Blood was added directly to the PCR reaction mix and amplified by real-time PCR. For each sample, the blood was tested with the screening primers to detect all species of *Plasmodium*. Examples of amplification and melt curves for each species, including blood spiked with gDNA from *Plasmodium knowlesi*, are shown in Figure 2. To evaluate the sensitivity and dynamic range of the assay, gDNA isolated from a patient sample infected with *Plasmodium falciparum *(9.2% parasitaemia) was serially diluted and spiked into uninfected blood (Figure 3). Amplification was detected in triplicate reactions across a range of 5 log dilutions with product confirmation by melt curve analysis. Based on the Ct values from the serial dilutions, the efficiency of the PCR directly from blood was calculated to be 84%.

**Figure 4:**
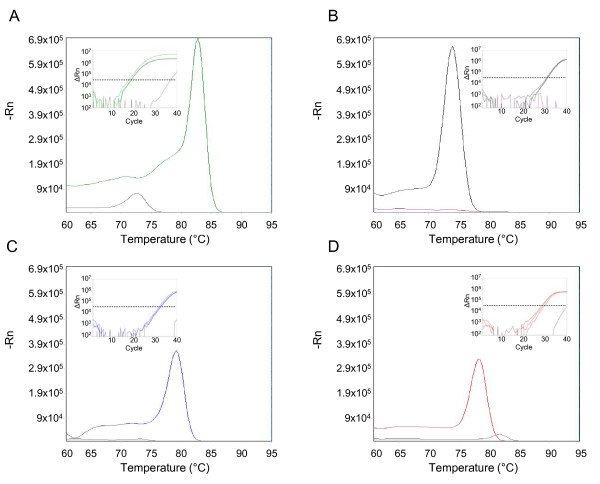
**Direct species identification from clinical samples**. (A-D) Representative melt curves and amplification curves (inset) from clinical samples with *Plasmodium falciparum *(A), *Plasmodium malariae *(B), *Plasmodium ovale *(C), and *Plasmodium vivax *(D) following real-time PCR with species-specific primers. Amplification curves are shown in triplicate but single melt curves are shown for clarity. Negative controls (gray) are from uninfected blood. The stippled line marks the threshold for the amplification curves.

In a second set of experiments, blood was tested with primers specific for *Plasmodium falciparum*, *Plasmodium vivax*, *Plasmodium ovale *and *Plasmodium malariae*. Amplification with primers specific to the four major species of *Plasmodium *identified the species directly from blood (Figure 4). The melting temperature values (Tm) for each species differ due to the varying sizes of the PCR products for each reaction. Non-specific amplification was observed at later cycles in the negative control with the primer sets for *Plasmodium falciparum *and *Plasmodium vivax *but these products were clearly distinguished from the specific product by melt curve analysis. Furthermore, the curves from these non-specific products crossed the threshold after cycle 35 and would therefore be called negative.

### Detection from dried blood spots

**Figure 5:**
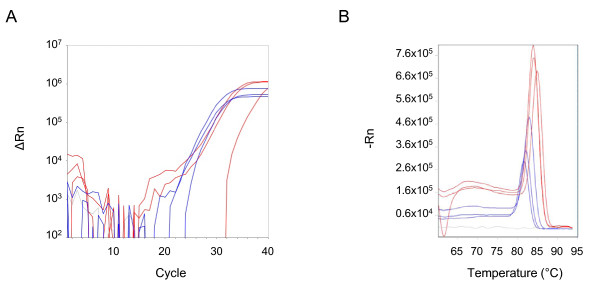
**Real-time PCR from dried blood spots**. Amplification curves (A) and melt curves (B) from DNA amplified directly from dried blood spots with the *Plasmodium *screening primers. Filter papers spotted with blood from a single patient sample infected with *Plasmodium falciparum *were added directly to the PCR master mix (blue) or washed first (red). Filter papers spotted with uninfected blood and added directly to the master mix serve as the negative control (gray). Samples were tested in triplicate.

An important application of this methodology is the direct amplification of *Plasmodium *DNA by real-time PCR from dried blood spots collected in the field. To evaluate this, blood spots were prepared from a clinical sample infected with *Plasmodium falciparum *(1.4% parasitaemia) and tested under the same reaction conditions used for whole blood with the *Plasmodium *screening primers. Spots excised from filter paper were either directly added to the PCR master mix or washed briefly in water. Amplification of *Plasmodium *DNA was detected in both the neat and washed filter papers, although the amplification curves for the neat samples had lower background and tighter replicates compared with the washed samples (Figure 5A). The melt curves for the neat and washed samples were very similar (Figure 5B). Given these results, neat blood spots were used in the subsequent validation experiments.

### Validation of the assay for *Plasmodium*

The sensitivity and specificity of this method for whole blood and blood spots was determined using blind panels of clinical samples (Table 1). For whole blood, the panel consisted of 100 samples: 67 from patients with suspected clinical malaria, 25 from asymptomatic refugees from endemic countries, 7 from volunteers with no history of malaria, as well as one sample with genomic DNA from *Plasmodium knowlesi *spiked into uninfected blood. These samples had been tested previously by real-time PCR performed on purified DNA using the method implemented at the Alberta ProvLab for malaria confirmation. This method uses a TaqMan probe and a different set of primers to screen for *Plasmodium *[10]. According to the results by real-time PCR on purified DNA, 64/67 samples from patients with suspected clinical malaria were positive, 9/25 of the asymptomatic refugee samples were positive, and the *Plasmodium knowlesi *sample was positive, giving a total of 74 positive and 26 negative samples in the panel. In the real-time PCR test directly from blood samples, 69 samples were positive and 31 were negative for *Plasmodium *(Table 1). The overall concordance of the real-time PCR results from blood and purified DNA was 95%. Five positives were undetected, resulting in an assay sensitivity of 93% and a specificity of 100%.

**Table 1:** Validation of real-time PCR from blood using blind clinical samples

		Raw blood* (n = 100)		Dried blood spots† (n = 48)	
		Pos	Neg	Pos	Neg
**Purified DNA**	Pos	69	5	38	0
	Neg	0	26	0	10

*Confirmed by real-time PCR according to Shokoples *et al *[10]

†Confirmed by nested PCR according to Snounou *et al *[12]

Microscopy results were available for 95/100 samples, 57 of which were positive with parasitaemias ranging from < 0.01% to 9.2%. Of these, 25 samples had an undetermined parasitaemia < 0.1%. Four of the false negative samples in the direct blood panel were from asymptomatic refugees and the fifth was from an acute malaria sample. All five samples were negative by microscopy.

For dried blood spots, patient samples collected in the field in Colombia were tested in a blind panel that consisted of 38 positives and 10 negatives (Table 1). The identity of the samples was confirmed by nested PCR on purified gDNA extracted from the blood spots performed in Colombia using the method of Snounou *et al *[12]. The panel was tested by adding the filter paper spots directly into the real-time PCR master mix. The concordance of the assay with nested PCR on purified DNA was 100%. The sensitivity and specificity of the assay were also 100%.

## Discussion

This report describes a methodology for DNA amplification directly from blood that is compatible with fluorescence detection by real-time PCR. Although this method cannot be used directly in the field, it facilitates the analysis of blood samples collected in field studies. Clinical trials on antimalarial or vaccine efficacy and prevalence or surveillance studies are just a few examples of applications of this test for high throughput analysis of blood samples. Either blood spots or whole blood samples can be transported to the lab and immediately tested by real-time PCR without the need for DNA extraction.

This method is particularly suited to studies seeking a rapid confirmation of malaria. The sensitivity is similar to real-time PCR performed on purified DNA and specificity is achieved through primer design to reduce non-specific amplification. At the Alberta ProvLab, malaria diagnosis is confirmed by a TaqMan real-time PCR assay that uses a previously published primer set for the *Plasmodium *screening reaction [16]. The primers from this assay were initially tested in the assay described here for real-time PCR from blood. Although amplification of *Plasmodium *DNA was achieved with these primers with good sensitivity, sporadic non-specific amplification was observed in the negative controls. Use of the primers recently published by Kamau et al. [13] reduced the formation of non-specific products. For some of the species identification reactions, improvements to the primer design may further reduce non-specific products that are detected as very late amplification curves. Furthermore, primers can be designed to produce amplicons with different melting curves for each species which would enable the species to be identified in a single multiplex reaction and further reduce the cost per test.

In general, the background fluorescence in this assay is higher than in other real-time PCR assays; the high concentration of SYBR^® ^Green required to overcome the quenching effect from the blood results in higher background fluorescence and the threshold was set at 20,000 or greater for these assays. With blood spots, fluorescence from the filter paper itself resulted in even higher background. Therefore, preliminary testing with blank filter papers and spots of uninfected blood is highly recommended to define the Tm and expected melt curve for a positive sample relative to background. Once this is established, the interpretation of results is straight-forward. Another important consideration is that the real-time PCR must be performed in tubes and not capillaries. Preliminary testing in capillaries on the LightCycler instrument from whole blood samples failed to generate amplification curves. It is hypothesized that during the course of the PCR, dried blood accumulates along the walls of the capillaries and blocks the emission of fluorescence.

In addition to the detection of *Plasmodium *DNA in blood, the reagents for direct PCR can also support the detection of other blood-borne pathogens. These reagents have previously been used to detect herpes simplex virus 2 and other viruses spiked into blood [8]. In malaria-endemic countries, the diagnosis of non-malarial causes of fever is critical to prevent morbidity and mortality, particularly from bacterial infections. A recent meta-analysis identified *Streptococcus pneumoniae*, *Salmonella enterica*, *Staphylococcus aureus*, *Escherichia coli *and *Haemophilus influenzae *as the major causes of bloodstream infections in African children [17]. The PCR test described here could be developed to detect these other organisms directly from blood samples.

In addition to the practical use of this assay for the qualitative analysis of field samples, the ability to perform real-time PCR for malaria directly from blood can be exploited in new technologies to bring molecular diagnostics for malaria to the point-of-care [18]. A number of technologies have been reported but they generally require an upstream module for DNA extraction [3,19,20]. Using the methodology described, this step can be omitted and samples can be immediately processed for diagnostic testing.

## Conclusions

The methodology described here supports the amplification of gDNA by fluorescence-based real-time PCR directly from blood samples infected with malaria. The advantage of this assay is the elimination of the DNA extraction step, thereby facilitating high-throughput analysis of samples collected in the field using state-of-the-art tools for the detection and characterization of *Plasmodium *infections.

## Competing interests

The authors declare that they have no competing interests.

## Authors' contributions

BJT and KAM performed the real-time PCR testing of blood samples and contributed equally to this work. AM provided dried blood spots from samples collected in the field and EA and OMA tested these samples by nested PCR. SKY and BJT designed the experiments and SKY wrote the manuscript. All authors read and approved the final manuscript.
